# Circulating miR-106a is a Novel Prognostic and Lymph Node Metastasis Indicator for Cholangiocarcinoma

**DOI:** 10.1038/srep16103

**Published:** 2015-11-04

**Authors:** Qingbao Cheng, Feiling Feng, Lumin Zhu, Yanhua Zheng, Xiangji Luo, Chen Liu, Bin Yi, Xiaoqing Jiang

**Affiliations:** 1 Department of Biliary Surgery I, Eastern Hepatobiliary Surgery Hospital, Second Military Medical University, 225 Changhai Rd, Yangpu, Shanghai, China; 2 Department of Hepatobiliary Surgery, The 404 Hospital of PLA, 8 Baoquan Rd, Weihai, Shandong, China; 3 Department of Blood Transfusion, Eastern Hepatobiliary Surgery Hospital, Second Military Medical University, 225 Changhai Rd, Yangpu, Shanghai, China

## Abstract

Cholangiocarcinoma (CCA) is a common biliary malignancy. Despite continuing advances, novel indicators are urgently needed to identify patients with a poor prognosis. Several microRNAs (miRNAs) have been reported to be dysregulated in CCA tissues. The purpose of the current study was to explore the potential use of certain miRNAs as serum indicators. A total of 157 individuals, including103 CCA patients, were recruited into this study. We first used qRT-PCR to evaluate 5 CCA-related miRNAs in the serum of 95 individuals to identify significantly deregulated miRNAs. A logistic regression was used to analyse the potential variables influencing lymph node metastasis. Cox proportional hazards regression models were applied to determine the association between possible prognostic variables and overall survival (OS). We observed that decreased serum miR-106a confers a higher likelihood of lymph node metastasis [hazard ratio (HR) 18.3, 95% confidence interval (CI) 5.9–56.4, *p* < 0.01]. Additionally, lower circulating miR-106a levels (HR 5.1; 95% CI 2.2–11.8; *p* < 0.01) and non-radical surgery (HR 4.2; 95% CI 2.3–7.7; *p* < 0.01) were independent predictors for poor prognosis. Together, reduced expression of serum miR-106a is a powerful prognostic indicator for CCA patients. The dismal outcome of these CCA patients might correlate with a higher risk of lymph node metastasis.

Cholangiocarcinoma (CCA) is a malignant tumour originating from the bile duct epithelium, and it frequently metastasizes to the lymph node. The morbidity associated with CCA has risen in recent years, but the pathogenesis mechanism and its predisposing factors remain unclear^1^. The only potentially curative treatment for CCA is radical resection^2^. Because of its lack of early symptoms, when clinical symptoms appear, most patients have reached an advanced stage, and radical resection is not a viable option^3^,^4^. Unfortunately, to date there are no definite sensitive and specific indicators for the early diagnosis of CCA^5^. The prognosis of CCA patients is dismal, usually measured by months, with death generally resulting from tumour metastasis^6^. In a retrospective analysis performed in our department, 40.4% of CCA patients developed lymph node metastasis, which was an independent prognostic predictor^7^. However, little is known regarding the exact molecular mechanisms underlying lymph node metastasis. In the clinical setting, the serum level of carbohydrate antigen 19-9 (CA19-9) is a marker that is frequently used for diagnosis and prognosis prediction in CCA patients. Unfortunately, CA19-9 levels are neither very sensitive nor particularly specific. Therefore, there is an urgent need to identify new indicators that will facilitate the identification of patients with a poor prognosis and permit adjuvant therapy for patients with a high risk of metastasis. In addition, with numerous chemotherapeutic drugs aimed to treat CCA, dynamic molecular indicators in the blood would be ideal to isolate CCA cohorts and monitor the potential benefits and side effects of different treatments.

MicroRNA (miRNA) is a type of endogenous coding small molecular RNA that widely exists in eukaryotes^8^. The discovery that miRNA expression is frequently dysregulated in malignant tumours underpins their pivotal role both from a basic science perspective and for its clinical usefulness^9^. Various studies have shown that miRNAs play critical roles in the development of human cancers^10^,^11^,^12^,^13^,^14^,^15^,^16^. In CCA tissues, several studies have identified some dysregulated miRNAs^13^,^14^,^17^. Some studies have indicated that miRNAs are also involved in lymph node metastasis^18^,^19^. Profiles of dysregulated miRNA isolated from plasma and serum have been generated and suggest that these miRNAs have diagnostic potential for human disease^20^,^21^. Serum circulating miRNAs are promising indicators for CCA for which the best chance of successful treatment is timely diagnosis and management; however, to date, few studies have specifically addressed the significance of circulating miRNAs in CCA patients. In the current study, we first performed a pooled analysis on the clinical validity of certain CCA-related miRNAs in 95 individuals to identify the specific miRNA as a dynamic indicator. The CCA cohort was extended to 103 cases for further clinicopathological and prognostic investigation.

## Results

### Patient characteristics

A total of 157 individuals including 103 CCAs, 34 benign bile-duct diseases (BBDDs) controls and 20 healthy controls were recruited into this study (Table 1). There were no significant differences in age (Student’s *t*-test) or gender (Pearson χ2 test) between cases and controls. In the CCA cohort, 60 patients (58.3%) acquired R0 resection. Overall survival (OS) was 74.8% at 1 year and 26.2% at 3 years. OS and recurrence rates for the R0 resections patients were 80.0% and 41.7% at 1 year, and 40.0% and 51.7% at 3 years, respectively. There were 52 (50.5%) patients confirmed dead and 15 (14.6%) patients confirmed with tumour recurrence at last follow-up. The mean age of patients was 58 years (range, 33 to 83). The median follow-up period (22.6 ± 27.1 months) was 26.9 months (range, 1 to 71 months). In addition, the CCA group and the other two control groups showed significant differences of T-Bil, CA-199, AST and ALT (*p* < 0.01). In CCA patients, 45 cases (43.7%) demonstrated lymph node metastasis.

### Indicator Selection and Validation in Serum Samples

**Figure 1:**
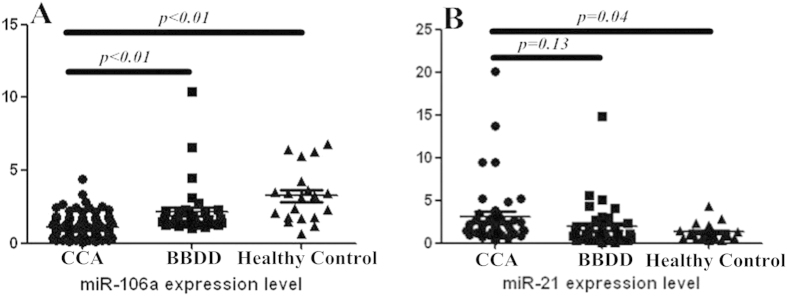
Expression analysis of miR-106a and miR-21 in the serum of patients with CCA, BBDD and healthy controls. (**A**) Serum miR-106a levels of CCA patients were significantly downregulated compared with those of BBDD patients and healthy controls; (**B**) MiR-21 levels in serum from patients with CCA were significantly elevated compared with healthy controls; however, the difference did not demonstrate significance compared with BBDD patients.

The goal of the present study was to explore the potential use of certain serum miRNAs as prognostic factors for CCA. First, a panel of 5 CCA-associated miRNAs was chosen on the basis of their reported relevance to CCA. Their expression levels were examined by RT-qPCR and quantitative PCR in 41 CCAs, 34 BBDDs and 20 healthy controls. The serum level of miR-106a was downregulated in CCA patients (1.27 ± 0.65) compare with BBDD patients (2.15 ± 1.80, *p* < 0.01) or healthy controls (3.27 ± 1.85, *p* < 0.01) using miR-16 as normalization control. Moreover, the serum level of miR-21 was higher in CCA patients (3.12 ± 3.80) than in BBDD controls (1.92 ± 2.72, *p* = 0.13) although the difference did not reach statistically significance. However, circulating miR-21 was significantly upregulated in CCA patients compared with healthy controls (1.29 ± 0.97, *p* = 0.04). The differences of serum levels of miR-224 and miR-224-2 were not significant among the three groups (*p* > 0.05). With regard to miR-370 the detection rates were <50% in all serum samples analysed by RT-qPCR; subsequently, the above three miRNAs (miR-224, miR-224-2, and miR-370) were excluded from further analytical studies. Of the two dysregulated miRNAs, the difference in the expression levels of miR-21 between CCA and BBDD patients did not achieve significance. On the basis of above results, we focused on miR-106a for its diagnostic and prognostic value. The CCA cohort was extended to 103 cases for further clinicopathological and prognostic analysis. The serum level of miR-106a was confirmed to be significantly downregulated in CCA patients (1.10 ± 0.77, *p* < 0.01). The results are shown in Fig. 1.

### The Diagnostic Value of miR-106a for CCA patients

**Figure 2:**
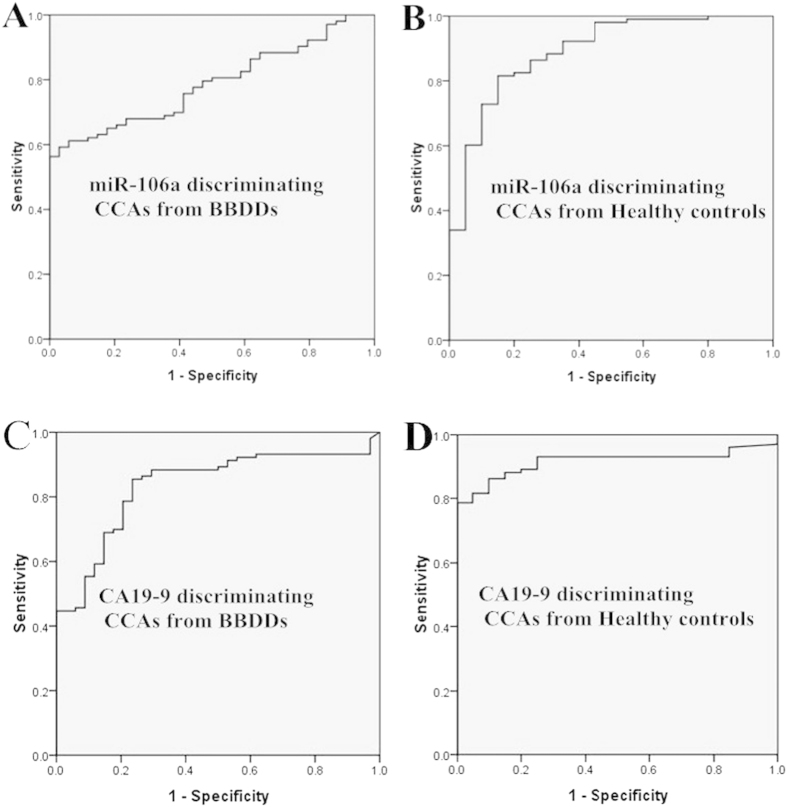
ROC curve analysis of serum miR-106a and CA19-9 for the diagnosis of CCA form BBDD or healthy controls. (**A**) AUC of serum miR-106a for discriminating CCA patients from BBDD patients; (**B**) AUC of serum miR-106a for discriminating CCA patients from healthy controls; (**C**) AUC of serum CA19-9 for discriminating CCA patients from BBDD patients; (**D**) AUC of serum CA19-9 for discriminating CCA patients from healthy controls.

ROC curve analyses were performed to evaluate the potential of serum miR-106a to distinguish CCA from BBDD patients and/or healthy controls. The AUC of serum miR-106a for discriminating CCA patients from BBDD controls was 0.79 (95% CI: 0.71–0.86; Fig. 2A). At the cut-off value of 1.00, the sensitivity and specificity were 56.3% and 100%, respectively, and the positive and negative likelihood ratios were 1.68 and 0.04, respectively. The AUC of serum miR-106a for discriminating CCA patients from healthy controls was 0.89 (95% CI 0.81–0.97; Fig. 2B). At the cut-off value of 1.68, the sensitivity and specificity for this marker were 81.6% and 85.0%, respectively, and the positive and negative likelihood ratios were 1.76 and 0.09, respectively. As a control variable, the AUC of serum CA19-9 for discriminating CCA patients from BBDD controls was 0.84 (95% CI 0.76–0.91; Fig. 2C), and at the cut-off value of 34.2, the sensitivity and specificity for this marker were 85.4% and 86.5%, respectively. The AUC of serum CA19-9 for discriminating CCA patients from healthy controls was 0.92 (95% CI 0.87–0.97; Fig. 2D), and at the cut-off value of 57.1, the sensitivity and specificity for this marker were 78.6% and 100.0%, respectively. Based on the above results, we conclude that the diagnostic value of miR-106a is moderate and superior to serum CA19-9.

### Serum miR-106a level and clinicopathological factors

**Figure 3:**
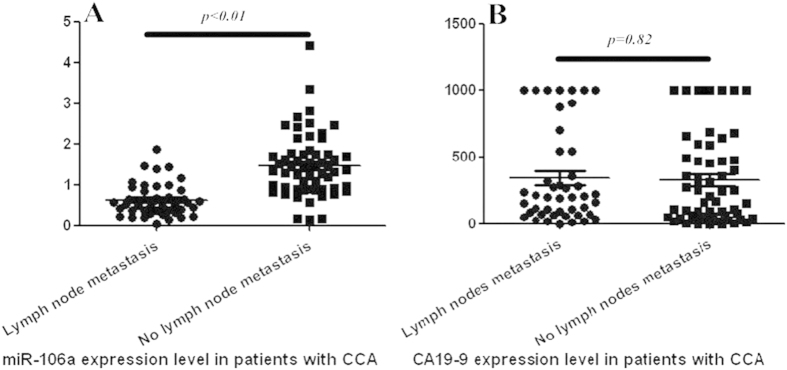
Expression analysis of miR-106a and CA19-9 in the serum of patients with CCA subdivided by metastasis to lymph node. (**A**) miR-106a (**B**) CA19-9.

CCA has the biological property of metastasis to regional lymph nodes in its early stage. It is well known that the expression levels of certain miRNAs are associated with clinicopathological variables in several cancers. As shown in Fig. 3A, serum miR-106a expression levels in CCA patients with lymph node metastasis were significantly decreased compared with those without metastasis (0.62 ± 0.40 vs.1.48 ± 0.78, respectively, *p* < 0.01), indicating that lower miR-106a levels might contribute to the lymph node metastasis of CCA. In contrast, no significant difference of serum CA19-9 levels was observed between these two groups (340.2 ± 352.8 vs. 326.9 ± 338.2, respectively, *p* = 0.82; Fig. 3B). Therefore, we examined the association between the expression level of circulating miR-106a and clinicopathological characteristics in 103 CCA patients. We defined the miR-106a level as ‘high expression’ when it was higher than a cut-off value of 1. The results are shown in Table 2. The circulating miR-106a level was significantly associated with lymph node metastasis (*p* < 0.01). To further investigate whether circulating miR-106a can serve as a predictor of lymph node metastasis, we performed multivariate logistic regression analysis, including serum miR-106a, CA19-9 level, tumour differentiation, neural invasion, p53, and MUC1 expression. Circulating miR-106a was identified as the only independent predictor of lymph node metastasis [hazard ratio (HR) 18.3, 95% confidence interval (CI) 5.9–56.4, *p* < 0.01].

### Down-regulation of miR-106a in serum samples was associated with poor prognosis in CCA patients

**Figure 4:**
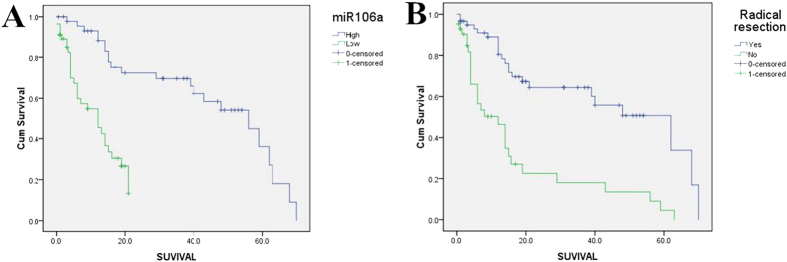
Kaplan-Meier survival curves of patients with CCA subdivided by serum miR-106a levels or radical resection. (**A**) miR-106a (**B**) Radical resection.

As the serum expression of miR-106a was significantly reduced in CCA patients, we explored the association between miR-106 serum levels with survival time. Initially, the median miR-106a serum level was utilized to divide the CCA patients into high and low groups by the cut-off value of 1.00 (miR-106a high, n = 46; miR-106a low, n = 57). The mean OS time for the entire CCA cohort was 32.8 ± 3.1 months. The miR-106a low expression group exhibited a shorter OS (*p* < 0.01, Fig. 4A). A Kaplan-Meier analysis also indicated that radical resection (*p* < 0.01, Fig. 4B) and no lymph node metastasis (*p* < 0.01) were associated with a longer OS. In contrast, age, gender, serum CA19-9 level, tumour diameter, tumour differentiation, p53, MUC1, and nerve invasion did not manifest a significant impact on OS. The detailed results are shown in Table 3. The mean OS time was 11.4 ± 1.2 months for patients with serum miR-106a level <1.00 compared with 45.0 ± 3.8 months for patients with serum miR-106a level >1.00. In addition, patients who received a radical resection had a mean OS time of 43.7 ± 4.1 months, while patients who did not had a mean OS time of 17.4 ± 3.7 months. Patients with lymph node metastasis had a mean OS time of 17.7 ± 3.8 months compared with 40.5 ± 3.8 months for the patients without.

Factors that were demonstrated to be significant in the univariate analysis entered a Cox hazard model to confirm the independent impact on OS. Based on multivariate analysis, low serum miR-106a level was identified as an independent prognostic factor for OS (HR 5.1; 95% CI 2.2-11.8; *p* < 0.01), which was the strongest factor among indices (Table 4). Radical resection also demonstrated independent influence on OS time (HR 4.2; 95% CI 2.3-7.7; *p* < 0.01). However, lymph node metastasis did not maintain a significant influence on OS time in multivariate analysis (HR1.4; 95% CI 0.7-2.8; *p* = 0.38). This influence was not independent in this series likely because of the colinearity between lymph node metastasis and circulating miR-106a levels.

## Discussion

The initial purpose of our work was to identify a set of miRNAs differentially expressed in healthy, BBDD, and CCA patients, that may aid in diagnosis and prognosis evaluation. Beginning with a pool of miRNAs, miR-106a manifested a moderate diagnostic value for CCA although the sensitivity and specificity were inferior to CA19-9. Our results supported that lower serum miR-106a levels were associated with higher risk of metastasis to lymph node. Additionally, we identified that circulating miR-106a was a prognostic predictor for OS, and a higher serum miR-106a level demonstrated a 33.6-months survival benefit in the current cohort. The overall mean survival time for the entire series was 32.8 ± 3.1 months, consistent with results reported in previous studies^7^,^22^,^23^,^24^. Based on these results, we believe that higher serum miR-106a level is strongly associated with a significantly better survival. This advantage might be attributed to less opportunity to metastasis to lymph nodes. From a clinical perspective, our study showed that the preoperative serum miR-106a level was an independent variable for predicting lymph node metastasis and prognosis evaluation in CCA patients. Moreover, this study confirmed the independent prognostic power of radical resection consistent with results reported previously^7^,^22^,^24^. In contrast, serum CA19-9 level, tumour differentiation, p53 protein expression, MUC1 protein expression, and nerve invasion demonstrated little prognostic value in the current cohort.

A number of studies have identified the stability of miRNAs in serum; therefore serum circulating miRNAs may become non-invasive and specific molecular diagnostic or prognostic markers for human diseases^20^,^25^. Circulating miRNAs have been postulated as novel biomarkers or indicators for various cancers^26^,^27^,^28^,^29^,^30^. In CCA patients, various miRNAs have been detected in tissues. MiR-106a and miR-21 have been indicated to be upregulated in CCA tissues^14^. After measuring plasma levels by qRT-PCR, Tomoya and colleagues suggested that miR-21 was upregulated in CCA patients and suggested that it could be used as a diagnostic biomarker for CCA^31^. In the current series, we confirmed that miR-21 was elevated in CCA patient serum compared with healthy controls, but the expression difference between CCA patients and BBDDs did not achieve significance. However, the expression levels of circulating miR-106a demonstrated a significant difference not only between CCAs and healthy controls but also between CCAs and BBDDs.

MiR-106a is a member of the miR-106a-363 cluster, located on chromosome X in humans^32^. MiR-106a plays an important role in the tumorigenesis of several human malignancies^33^,^34^,^35^,^36^,^37^. Chen *et al.*^14^ determined that miR-106a was increased by 110-fold in CCA tissues. MiR-106a was also found to be overexpressed in gastric cancer^38^, colorectal cancer^39^,^40^, and pancreatic cancer^37^. However, miR-106a was found to be down-regulated in glioma and play tumor suppressor role^41^. Only recently higher miR-106a tissue levels have been described to be associated with glioma invasion by targeting metalloproteinases-2^34^.

Lymph node metastasis is a dependent prognostic factor as confirmed in the current series. However, this influence was not independent in this series likely because of the colinearity between lymph node metastasis and circulating miR-106a level. Interestingly, we found that the circulating miR-106a level significantly correlated with lymph node metastasis. Patients with a lower serum miR-106a level conferred more opportunity to lymph node metastasis (HR 18.3, 95% CI 5.9-56.4, *p* < 0.01). In addition, a low circulating miR-106a level was confirmed to be an independent poor prognostic predictor (HR 5.1; 95% CI 2.2-11.8; *p* < 0.01). Several reports have confirmed the prognostic value of miR-106a in astrocytoma^42^, glioblastoma^43^, and gastric carcinoma^44^. The downregulation of circulating miR-106a in the current patient group is not consistent with findings drawn from the CCA tissues. This inconsistency may be explained by the non-secreting nature of miR-106a and likely the effect of the tumour microenvironment. The source of circulating miRNAs has been investigated by several studies but is still a source of debate. Elhelw and colleagues argued that serum miRNA levels not only were a result of tumours, but also maybe be a result of the immune response^45^. This discrepancy has been demonstrated by several reports, such as miR-195 in breast cancer^46^,^47^ and miR-122^48^,^49^,^50^ and miR181a^51^,^52^ in hepatocellular carcinoma. Notably, miR-106a has recently been implicated in chemotherapy resistance. Circulating miR-106a was indicated to be upregulated in non-responders after oxaliplatin-based chemotherapy in metastatic colorectal cancer patients and as a biomarker to predict outcome^53^. In ovarian cancer, Huh and colleagues published an article and suggested upregulated miR-106a was associated with paclitaxel resistance^54^. However, the mechanisms of these results have not been addressed, and this characterization is fundamentally necessary to acquire a deeper understanding of cancer progression.

Taken together, these findings demonstrate that serum miR-106a level is downregulated in CCA patients and associated with metastasis to lymph node and prognosis. Higher circulating miR-106a level confers a survival benefit. A lower miR-106a level confers a higher risk for lymph node metastasis in CCA patients. The diagnostic value of miR-106a for CCA patients is moderate. Collectively, these results indicate that miR-106a presents a clinically promising indicator that can facilitate lymph node metastasis risk assessment and prognosis evaluation in CCA patients. These findings require large-scale prospective validation.

## Methods

The methods were performed in accordance with the approved guidelines.

### Study Design and Patients

This study was approved by ethics boards of Eastern Hepatobiliary Surgery Hospital, and informed consent was obtained from all subjects recruited. No attempt was made to define a target statistical power. From February 2010 through January 2014, we prospectively enrolled a cohort of individuals, including CCA patients (n = 103) who underwent resection with a curative intent, 34 BBDD patients (20 primary bile duct stone and 14 congenital biliary duct cyst patients) and 20 healthy controls. The distribution of gender and age was not significantly different between patients and healthy controls. All CCA patients were required to have histologically confirmed adenocarcinoma. According to the AJCC 7th TNM stage system, there were 76 peri-hilar CCAs and 27 extrahepatic CCAs in this cohort. Tumour stage was determined according to the AJCC 7th TNM stage. In current study, the peripheral-blood samples (fasting) were drawn from all patients preoperatively. None of the patients had received prior treatment, in particular, chemotherapy or radiotherapy for CCA patients, and none suffered from any tumours or any relevant critical illnesses. CA19-9 levels in serum samples were measured by standard enzyme immunoassay as a routine clinical test. After resection, tissue samples were examined histopathologically by at least two pathologists. Routine analyses were performed on all CCA specimens (pathological grade, lymph node metastasis, nerve invasion, vascular invasion, and immunostaining for p53, MUC1, CK19 and CA19-9). Complete clinicopathological data were collected for each patient. The data regarding the subjects were obtained from medical records, pathology reports and personal interviews with the subjects. The data collected included age, gender, total bilirubin level (T-Bil), alanine aminotransferase (ALT) level, aspartate transaminase (AST) level, CA19-9 level, and lymph node metastasis status according to previous surgical operative notes. As a control, serum samples were drawn from 20 healthy subjects confirmed through comprehensive medical examination in Changzheng Hospital (Shanghai, China). The comparative baseline clinical characteristics of CCA, BBDD patients, and healthy controls are described in Table 1.

### Selection of circulating miRNAs as candidate markers

According to previous reports^11^,^13^,^14^, a group of most CCA-associated miRNAs, including miR-21, miR-106a, miR-224, miR-224-2, and miR-370, was selected to evaluate their potential as circulating indicators for CCA. These miRNAs were previously found to be differentially expressed in CCA tissues and normal bile duct mucosa. Although multiple miRNAs with dysregulated expression in CCA have been discovered, we focused on these miRNAs because they have been reported as the most dramatically dysregulated. Using miR-16 as normalization control, the potential miRNAs markers chosen were verified on serum samples through reverse transcription (RT) and quantitative PCR. The diagnostic efficacy and correlation with lymph node metastasis and survival of CCA patients were analysed. Serum preparation, RNA extraction, reverse transcription (RT) and quantitative PCR (qPCR) procedures have been previously described^55^. RT and qPCR kits allowed for accurate miRNA analysis (Applied Biosystems) and were used to evaluate the expression of the chosen miRNAs.

### CCA patient follow-up

CCA patients were followed up every 3 months. All CCA patients were prospectively monitored by serum carcinoembryonic antigen (CEA), CA19-9, abdomen ultrasonography with an interval of 1 month during the first year postpoperatively. A computed tomography scan of the abdomen was performed every 3 months. If recurrence was suspected, a computed tomography scan or magnetic resonance imaging was performed immediately. Most causes of death were recurrence and metastasis. Patients with confirmed recurrence received further treatment, which was mainly based oral tegafur chemotherapy and external radiotherapy. Otherwise, symptomatic treatment was provided. Follow-up was terminated on May 6, 2015. OS was defined as the interval elapsing between the date of surgery and date of death or censoring at the most recent follow-up.

### Statistical Analyses

Statistical analyses were performed using SPSS Statistical Software version 17.0 (SPSS, Inc.). Because of the magnitude and range of relative miRNAs expression levels observed, the results were log transformed for the analysis. There was no evidence against normality for the log transformed data as confirmed using the Kolmogorov-Smirnov test. Descriptive statistics for quantitative variables are given as the mean ± standard deviation. The difference in quantitative variable was tested using Student’s *t*-test. The Pearson Chi-square test or Fisher’s exact test was used to compare qualitative variable. Receiver operating characteristics (ROC) curves were constructed and the area under the curve (AUC) was calculated to evaluate the sensitivity and specificity for predicting CCAs and BBDDs or healthy controls based on the expression level of dysregulated miRNAs. Logistic regression was used to analyse the potential variables influencing lymph node metastasis. Survival analyses were executed following the Kaplan-Meier method, and comparisons were made using the log rank test. Beginning with a pool of significant predictors identified in the univariate analyses, variables were evaluated in multivariable Cox proportional hazards models, including only variables with a *p* value < 0.05. Two-sided *p* values < 0.05 were considered significant.

## Additional Information

**How to cite this article**: Cheng, Q. *et al.* Circulating miR-106a is a Novel Prognostic and Lymph Node Metastasis Indicator for Cholangiocarcinoma. *Sci. Rep.* **5**, 16103; doi: 10.1038/srep16103 (2015).

## Figures and Tables

**Table 1:** Summary of clinical parameters of the enrolled individuals.

	CCA (n = 103)	BBDD (n = 34)	Healthy Control (n = 20)
Male n (%)	55 (53.3)	22 (64.7)	12 (60.0)
Age (median, range)	58 (33, 83)	45 (20, 78)	45 (19, 83)
Laboratory values (median, range)			
Tbil (μmol/L)	183 (9, 493)	78 (33, 135)*	11 (9, 15)*
AST (U/L)	94 (69, 212)	31 (27, 45)*	19 (16, 23)*
ALT (U/L)	101 (35, 368)	22 (13, 37)*	17 (14, 26)*
CA19-9 (U/ml)	205 (1, 1000)	42 (12, 56)*	23 (6, 31)*

^*^mean *p* < 0.01 compared with CCA group.

CCA: cholangiocarcinoma, BBDD: benign bile-duct disease, Tbil: total bilirubin, AST: aspartate transaminase, ALT: alanine aminotransferase, CA19-9: carbohydrate antigen 19-9.

**Table 2:** The correlation of circulating miR-106a with clinicopathological factors in CCA patients.

	Low expression	High expression	*p* value
Age (y)			
≲65	40	29	0.44
>65	17	17	
Gender			
Male	33	22	0.31
Female	24	24	
Serum CA19-9 level (U/ml)			
≤37	9	8	0.83
>37	48	38	
Radical resection			
Yes	30	30	0.20
No	27	16	
Well differentiation			
Yes	2	4	0.26*
No	55	42	
Lymph node metastasis			
Yes	39	6	<0.01
No	18	40	
Nerve invasion			
Yes	39	26	0.21
No	18	20	
p53			
Positive	22	15	0.53
Negative	35	31	
MUC1			
Positive	24	18	0.76
Negative	33	28	

^*^means result of Fisher exact test.

**Table 3:** Prognostic factors for survival by univariate analysis.

Factors	Patients (n)	Mean survival	Standard error	95% Confidence Interval (CI)	*p* value
Age (years)					
<65	67	39.4	3.4	26.4–42.1	0.51
≧65	36	24.6	5.1	20.9–39.9	
Gender					
Male	55	32.0	3.6	24.8–39.1	0.64
Female	48	36.3	6.2	24.1–48.4	
Serum CA19-9 level (U/L)					
≤37	17	40.2	7.5	25.4–55.0	0.39
>37	86	31.2	3.3	24.7–37.8	
Serum miR-106a level					
≤1	57	11.4	1.2	9.1–13.7	<0.01
>1	46	45.0	3.8	37.5–52.5	
Radical resection					
Yes	60	43.7	4.1	35.7–51.9	<0.01
No	43	17.4	3.7	10.2–24.6	
Neural invasion					
Yes	65	31.0	4.8	21.7–40.3	0.57
No	38	33.6	4.0	25.7–41.4	
Tumor diameter (cm)					
<3	48	34.2	4.2	26.0–42.6	0.31
≧3	55	32.0	4.5	23.2–40.7	
Lymph node metastasis					
Yes	45	17.7	3.8	10.2–25.2	<0.01
No	58	40.5	3.8	33.1–47.9	
Well differentiation					
Yes	6	29.2	8.8	12.1–46.4	0.93
No	97	32.8	3.2	26.5–39.0	
p53					
Positive	37	34.7	6.3	22.4–47.0	0.77
Negative	66	32.7	3.6	25.7–39.7	
MUC1					
Positive	42	26.9	4.2	18.6–35.2	0.20
Negative	61	36.9	4.2	28.6–45.2	

**Table 4:** Prognostic factors for survival by Cox proportional hazards model.

Independent factors	Hazard Ratio	95% CI	*p* value
low serum miR-106a level	5.1	2.2–11.8	<0.01
radical resection	4.2	2.3–7.7	<0.01
Factors evaluated:			
serum miR-106a level			
radical resection			
lmph node metastasis			
